# Combining Organic and Inorganic Wastes to Form Metal–Organic Frameworks

**DOI:** 10.3390/ma13020441

**Published:** 2020-01-17

**Authors:** Eléonore Lagae-Capelle, Marine Cognet, Srinivasan Madhavi, Michaël Carboni, Daniel Meyer

**Affiliations:** 1 ICSM, University Montpellier, CEA, CNRS, ENSCM, 30207 Marcoule, France; eleonore.lagae-capelle@cea.fr (E.L.-C.); marine.cognet@cea.fr (M.C.); daniel.meyer@cea.fr (D.M.); 2 School of Materials Science and Engineering, Nanyang Technological University, Singapore 639798, Singapore; madhavi@ntu.edu.sg; 3 Energy Research Institute @ NTU, ERI @ N, Nanyang Technological University, Singapore 639798, Singapore

**Keywords:** recycling batteries, recycling plastic bottles, metal–organic frameworks, selective precipitation, hydrometallurgy process

## Abstract

This paper reports a simple method to recycle plastic-bottle and Li-ion-battery waste in one process by forming valuable coordination polymers (metal–organic frameworks, MOFs). Poly(ethylene terephthalate) from plastic bottles was depolymerized to produce an organic ligand source (terephthalate), and Li-ion batteries were dissolved as a source of metals. By mixing both dissolution solutions together, selective precipitation of an Al-based MOF, known as MIL-53 in the literature, was observed. This material can be recovered in large quantities from waste and presents similar properties of purity and porosity to as-synthesis MIL-53. This work illustrates the opportunity to form hybrid porous materials by combining different waste streams, laying the foundations for an achievable integrated circular economy from different waste cycle treatments (for batteries and plastics).

## 1. Introduction

The development of batteries is one of the crucial main directions for the progress of renewable energies, and batteries have been extensively developed as mobile energy sources (e.g., for mobile phones and cars) [1]. Li-ion-battery (LiB) breakthrough technology has had the lion’s share of the development since the early 1990s due to the batteries’ high energy density and relative safety [2]. LiBs are mainly composed of a cathode, an anode, an electrolyte, and a separator. While the anode is a copper foil coated with a graphite-based material, the cathode is generally an aluminum electroactive-metal-layered material (e.g., based on Fe, Ni, Mn, or Co). The expected growth in the usage of LiBs along with the induced criticality of some metals (e.g., Co), the regulation of LiBs in several countries, and the toxicity of some battery compounds impose the need to develop efficient recycling processes with low environmental impact [3]. The current recycling processes of batteries are in an early stage of development with limited involvement in a circular economy and are ruled by regulation and the actual economic model. The main issues for future developments are usually the variability in the batteries’ composition and the difficulty involved in their collection and recovery from other wastes. Despite the expected criticality of Co, the metal resources needed for the synthesis of batteries are abundant and metal prices are still low (in 2019), posing the challenge for the development of a complex and efficient recycling process [4].

In another field, the recycling of plastic waste is also a major societal problem. Only 10% of all plastics produced are recycled, excluding those that are incinerated [5]. The difficulties in recycling plastic come from the plastics’ high variability of composition and their dispersion throughout the countryside. For example, plastic bottles are composed of a non-biodegradable polymer polyethylene terephthalate (PET), which makes up 10% of plastic production [6]. Downstream products from PET recycling are of low value, making the process (by physical or chemical treatment) not interesting when compared to the low price of virgin PET. Moreover, in addition to making no economic sense, the current recycling of PET has some environmental issues due to the presence of contaminants [7].

Metal–organic frameworks (MOFs) or hybrid porous organic/inorganic materials are a class of crystalline solids organized by the self-assembly of organic linkers with metal ions or clusters [8]. They have strong potential to be used for a wide range of applications due to their specific properties (such as stability, large porosity, or high surface area) [9]. The main issues of these materials remain their synthesis, generally at the gram scale, and the final price of the materials. Recently, these materials have been produced using commercial depolymerized PET as a source of organic ligands to build various MOFs, based on Zr [10,11], Cu [12], Zn [13], Cr [14,15], Al [14,16], Ga [14], and V [16], with a high purity. We have recently proposed the use of LiB waste as a source of metal ions for the synthesis of such materials [17,18,19]. We have developed a LiB recycling approach to obtain Al MOFs, Ni–Mn MOFs, and Cu MOFs directly from a rough, black mass dissolution solution by successive precipitation using benzene tricarboxylic acid.

**Figure 1:**
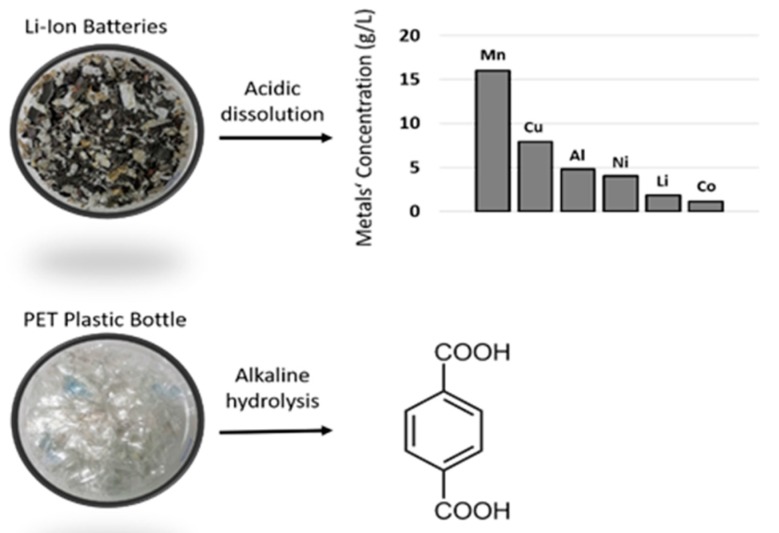
Dissolution of Li-ion batteries (**top**) and alkaline hydrolysis of plastic bottles (**bottom**).

By combining the PET and LiNMC (Ni, Mn, Co) waste streams, we have laid the foundation for an integrated recycling process, leading to potential high-value products (such as Al MOFs). The work was done on real waste—plastic bottles used for regular water and crushed lithium Mn–Ni (LMN) batteries—as depicted in Figure 1.

## 2. Materials and Methods 

### 2.1. Materials

Transparent PET water-bottle waste from various manufacturers (cristaline, evian and volvic, France) was recovered directly from trash cans, and crushed LiB waste, mainly composed of plastics, carbon, and metals, was provided by an industrial manufacturer and used without further physical treatment. Other reagents were purchased from Sigma and were used without any purifications.

A Bruker D8 Advance diffractometer (Bruker, Billerica, MA, USA) equipped with a Cu anode was used for PXRD analysis. SEM images of the materials were recorded with a FEI Quanta 200 environmental scanning electron microscope (ThermoFisher, Waltham, MA, USA) at an acceleration voltage of 30 kV under vacuum. A Mettler-Toledo TG (Mettler-Toledo, Greifensee, Switzerland) with an autosampler was used for TGA. An ASAP 2020 (Micromeritics, Norcross, GA, USA) at 77 K was used to measure the nitrogen uptake. The samples were outgassing at 200 °C for 24 h prior to the analysis, and the specific surface areas were determined with the BET method. A ^1^H NMR was recorded at room temperature using a 400 MHz spectrometer (Bruker, Billerica, MA, USA).

### 2.2. Protocols

Depolymerization of PET bottles: 5 g of plastic bottles (cut into pieces of around 2 cm) were introduced to an Erlenmeyer flask with 2.8 g of solid NaOH and 50 mL of ethylene glycol. The solution was slowly stirred and heated to 180 °C for 12 h. Then, distilled water was added, drop by drop, to solubilize the mixture and to obtain a clear solution (around 50 mL). 

Dissolution of Li-ion batteries: 15 g of crushed batteries were added to 150 mL of 2 N HCl solution. The solution was slowly stirred for 24 h. The plastics and carbon materials were removed by filtration and centrifugation and, finally, a clear metal ion solution was obtained. The solution was analyzed by ICP-OES (Figure 1). The concentrations (g/L) of the elements were 16.0 (Mn), 7.9 (Cu), 4.8 (Al), 4.0 (Ni), 1.8 (Li), and 1.1 (Co). The metal composition was in accordance with LMN-type batteries (i.e., Al and Cu were in high concentration and came from the contactors of the battery).

Selective precipitation of MOFs: 530 µL of the terephthalate solution (equivalent to 16 mg of ligand) was mixed with 1 mL of DMF (Dimethylformamide), and 1 mL of the battery-waste solution and the clear solution was heated to 70 or 90 °C for 12 h. A precipitate was observed after the solvothermal reaction and was separated by centrifugation. The powder was washed with DMF at 150 °C for one night and washed with H_2_O at 90 °C for 4 h before being dried at 90 °C for 12 h.

## 3. Results and Discussion

Plastic bottles and LiBs can be easily solubilized in alkaline and acidic solutions, respectively. The metal composition of the dissolution solution of batteries was determined by ICP-OES, whereas the ligand concentration in the dissolution solution of PET bottles was determined by ^1^H NMR. To determine and quantify the terephthalate concentration in this solution, an acidic solution of HCl (10 mL of a 30% HCl solution) was carefully added until the precipitation of a white powder occurred. The powder was centrifuged and washed with water and ethanol and then dried at 90 °C overnight. The organic product was then isolated, dried, and analyzed by ^1^H NMR (Figure S1). The peak at 8.04 ppm corresponded to the four aromatic protons of the terephthalate ligand and the peak at 13.27 ppm to the two COOH functionalities. The peak at 3.40 ppm corresponded to some residual ethylene glycol that came from the hydrolysis of the PET or from the solvent used during the depolymerization reaction. The concentration of ligands in the solution was, in this case, determined as 30 g/L.

In order to keep the process as simple as possible, quantities of both dissolution solutions were mixed together (carefully) and some DMF was also added to keep the mixture clear, with all components soluble in such conditions. The DMF was crucial to avoid the precipitation of the organic ligand directly after mixing both solutions. After heating at different temperatures (70 or 90 °C) for 12 h, a white precipitate appeared and was isolated and analyzed.

**Figure 2:**
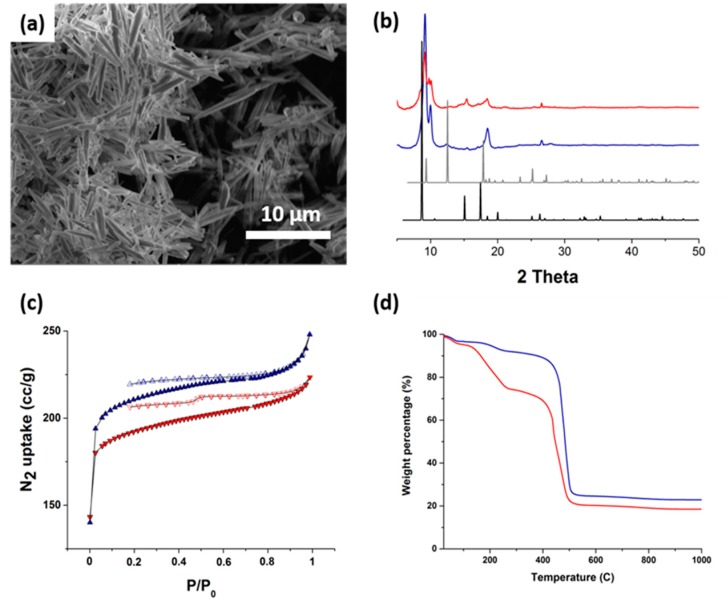
(**a**) SEM images of the material obtained at 90 °C; (**b**) PXRD; (**c**) BET and (**d**) TGA analyses for the materials obtained at 70 °C (red) and 90 °C (blue). The black and gray XRD patterns correspond, respectively, to the simulated XRD patterns of high-temperature and low-temperature phases of MIL-53 (Al).

SEM analysis revealed the formation of well-organized materials with a needle shape of around 10 to 5 µm depending on the temperature of the synthesis (90 and 70 °C, respectively) (Figure 2a and Figure S2). The materials obtained at 90 °C appeared more crystalline with thicker and longer needles than those obtained at 70 °C. The EDX measurements of both samples revealed that the materials were composed of Al, showing the selective precipitation of this metal (Tables S1 and S2).

The PXRD patterns of both compounds were very similar and were in accordance with the formation of a crystalline hybrid material (low-angle peaks with broad signals) (Figure 2b). The patterns corresponded to a previously reported MOF, named MIL-53 (Al) in the literature, based on the same ligands and Al as the metal node. An extra picture was also observed for the material obtained at 70 °C. This particular material was reported to be flexible, which depended on a stimulus (guest molecules) conducting a reversible change in the material’s crystalline structure (high-temperature and low-temperature phases). Here the obtained materials were a mixture of such phases with different guest molecules inside the pore cavities.

Permanent porosity of the MOFs was demonstrated by N_2_ adsorption at 77 K and revealed that the materials had a high BET surface area with values of 742 m^2^/g at 70 °C and 813 m^2^/g at 90 °C (Figure 2c). These two values are similar to but lower than those reported in the literature (around 1100 m^2^/g) for as-synthesized materials. 

TGA analyses were in accordance with a material with the composition of [Al^III^(OH)(BDC)] (Figure 2d). A first loss of between 10 and 30 wt.% (at 90 and 70 °C, respectively) was observed between 100 and 240 °C and was attributed to some residual solvent molecules (water and/or DMF). This occurred prior to the degradation of the framework at around 450 °C with the decomposition of the organic ligand to form the corresponding oxide material.

Finally, by mixing the different dissolution solutions corresponding to 5 g of crushed batteries (containing 240 mg of Al) and 3 g of plastic bottles (containing 1.8 g of the organic ligand), it was possible to recover around 2.5 g of Al MOF at 70 and 90 °C. By taking into account the formula of the MOF, [Al^III^(OH)(BDC)], the Al precipitation was total. After the removal of Al from the solution, it was not possible to precipitate other metals after adding more plastic-bottle dissolution solution for a second precipitation step in the same conditions. 

## 4. Conclusions

For the first time, to our knowledge, a high-quality Al-MOF-type porous material was formed using only two sources of waste—Li-ion batteries and PET plastic bottles. This work mainly focused on the production of one type of MOF. The production of other kinds of MOF (Cu, Ni, Mn, or Co based) may be considered in the near future. The obtained material, known as MIL-53 in the literature, has very similar properties to the material formed with pure chemical compounds. The main issues for the further development of the current approach are the replacement of DMF with a greener solvent and the rationalization of its energy use (i.e., decreasing the temperature). In addition to the low cost of MOF synthesis, the proposed coupling of two kinds of independent waste streams will provide other options for the development of recycling processes.

## Supplementary Materials

The following are available online at https://www.mdpi.com/1996-1944/13/2/441/s1: Figure S1: ^1^H NMR spectra obtained after the alkaline treatment of the PET plastic, Figure S2: SEM image of the material obtained at 70 °C, Table S1: EDX analysis of the material obtained at 70 °C, Table S2. EDX analysis of the material obtained at 90 °C.
