# Supplementary material for: Combining Organic and Inorganic Wastes to Form Metal–Organic Frameworks
Source: Materials (Basel). 2020 Jan 17;13(2):441. doi: 10.3390/ma13020441 (PMC7013536; doi:10.3390/ma13020441)
Supplement: Supplementary file 1 [file materials-13-00441-s001.pdf]

materials

Supporting Information for

Combining organic and inorganic wastes to form Metal-Organic Frameworks

Eléonore Lagae-Capelle [1], Marine Cognet [1], Srinivasan Madhavi [2,3], Michaël Carboni [1,*] and Daniel Meyer [1]

[1] ICSM, University Montpellier, CEA, CNRS, ENSCM, Marcoule 30207, France; eleonore.lagae-capelle@cea.fr (E.L.C.); marine.cognet@cea.fr (M.C.); daniel.meyer@cea.fr (D.M.)
[2] School of Materials Science and Engineering, Nanyang Technological University, Singapore 639798; madhavi@ntu.edu.sg
[3] Energy Research Institute @ NTU, ERI @ N, Nanyang Technological University, Singapore 639798, Singapore
* Correspondence: michael.carboni@cea.fr; Tel.: +33-466-339-204

Table of content

1. 1H NMR spectra obtained after alkaline treatment of bottles S2
2. SEM image of the material obtained at 70 °C S2
3. Table S1: EDX analysis of the material obtained at 70 °C S3
4. Table S2: EDX analysis of the material obtained at 90 °C S3

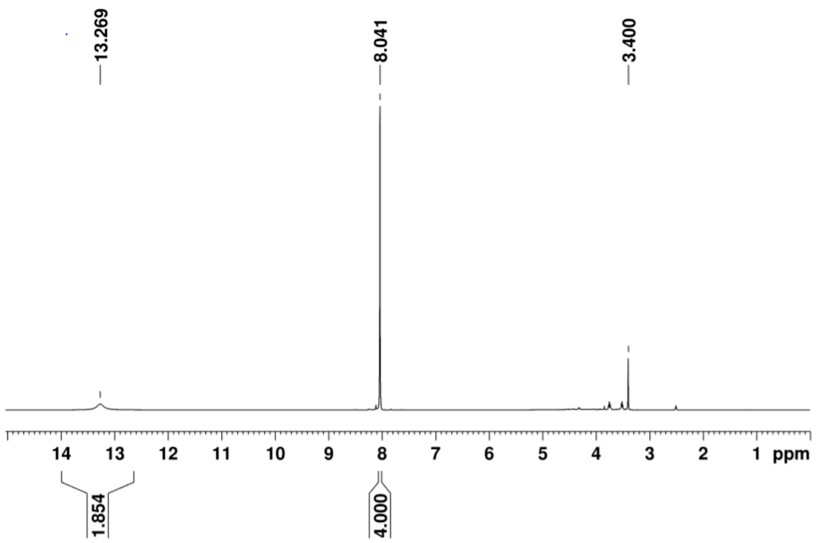

Figure S1. ^{1}H NMR spectra obtained after alkaline treatment of PET plastic bottles.

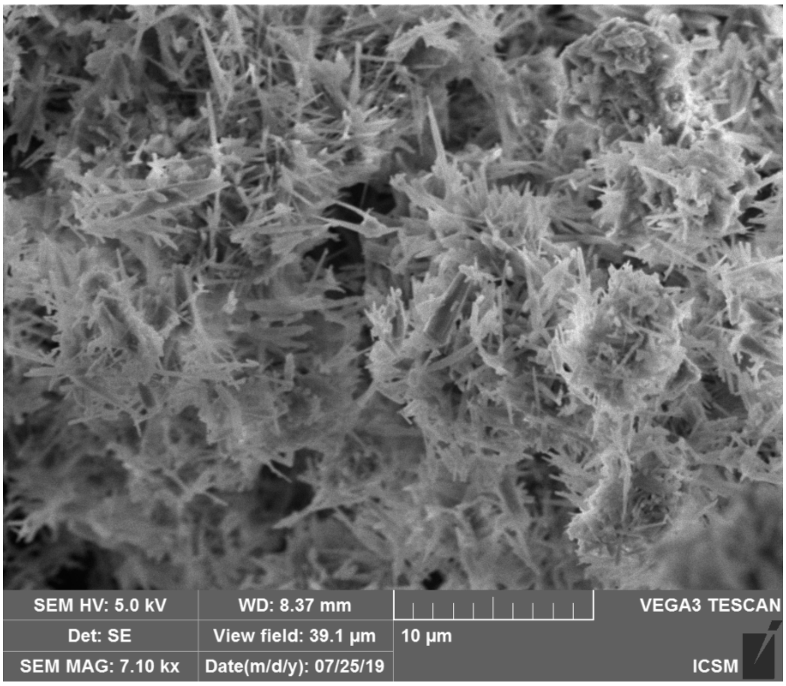

Figure S2. SEM image of the material obtained at 70 °C.

Table 1. EDX analysis of the material obtained at 70 °C.

Element	A	Raie	Mass. [%]	Comp. [%]	Comp. norm. [%]
Oxygen	8		11.8377561	0	0
Aluminium	13	K-Serie	12.8033783	24.1915183	92.6733453
Manganese	25	L-Serie	0.56895085	0.90033794	3.44903233
Cobalt	27	L-Serie	0.01020836	0.01297976	0.04972311
Nickel	28	L-Serie	0.0286284	0.0364323	0.13956557
Copper	29	L-Serie	0.85515171	0.96280533	3.68833364
Total			26.1040737	26.1040737	100

Table 2. EDX analysis of the material obtained at 90 °C.

Element	A	Raie	Mass. [%]	Comp. [%]	Comp. norm. [%]
Oxygen	8	K-Serie	9.98592426	0	0
Aluminium	13	K-Serie	11.0726007	20.921277	44.7604821
Manganese	25	L-Serie	0.200058	0.31658238	0.67731907
Cobalt	27	L-Serie	0.01170343	0.01488072	0.03183689
Nickel	28	L-Serie	0.00027621	0.0003515	0.00075203
Copper	29	L-Serie	0.13878206	0.1562531	0.3342991
Total			46.7405087	46.7405087	100
